# Aniline Induces Oxidative Stress and Apoptosis of Primary Cultured Hepatocytes

**DOI:** 10.3390/ijerph13121188

**Published:** 2016-11-30

**Authors:** Yue Wang, Hong Gao, Xiao-Lin Na, Shu-Ying Dong, Hong-Wei Dong, Jia Yu, Li Jia, Yong-Hui Wu

**Affiliations:** 1 Department of Occupational Health, College of Public Health, Harbin Medical University, Harbin 150086, China; wangyue5056@163.com (Y.W.); donghongwei0824@163.com (H.-W.D.); 2 Department of Sanitary Microorganisms, College of Public Health, Harbin Medical University, Harbin 150086, China; m15114515383@163.com; 3 Department of Environmental Health, College of Public Health, Harbin Medical University, Harbin 150086, China; jljz5858@163.com (X.-L.N.); dong.shuying@163.com (S.-Y.D.); jiali1013@126.com (J.Y.)

**Keywords:** aniline, apoptosis, reactive oxygen species, hepatocytes

## Abstract

The toxicity and carcinogenicity of aniline in humans and animals have been well documented. However, the molecular mechanism involved in aniline-induced liver toxicity and carcinogenesis remains unclear. In our research, primary cultured hepatocytes were exposed to aniline (0, 1.25, 2.50, 5.0 and 10.0 μg/mL) for 24 h in the presence or absence of *N*-acetyl-l-cysteine (NAC). Levels of reactive oxygen species (ROS), malondialdehyde (MDA), and glutathione (GSH), activities of superoxide dismutase (SOD) and catalase (CAT), mitochondrial membrane potential, DNA damage, cell viability, and apoptosis were detected. Levels of ROS and MDA were significantly increased and levels of GSH and CAT, activity of SOD, and mitochondrial membrane potential in hepatocytes were significantly decreased by aniline compared with the negative control group. The tail moment and DNA content of the tail in exposed groups were significantly higher than those in the negative control group. Cell viability was reduced and apoptotic death was induced by aniline in a concentration-dependent manner. The phenomena of ROS generation, oxidative damage, loss of mitochondrial membrane potential, DNA damage and apoptosis could be prevented if ROS inhibitor NAC was added. ROS generation is involved in the loss of mitochondrial membrane potential and DNA injury, which may play a role in aniline-induced apoptosis in hepatocytes. Our study provides insight into the mechanism of aniline-induced toxicity and apoptosis of hepatocytes.

## 1. Introduction

The aniline production worldwide surpassed 5.6 million tons in 2016 [1] and its industrial use is mainly in the manufacture of dyes, pigments, herbicides, fungicides, explosives, isocyanates, hydroquinones, and rubber chemicals. A large number of individuals have a risk of exposure to aniline through air, water and diet. Acute exposure to aniline can induce methemoglobinemia, hemolysis, and hemolytic anemia [2,3,4]. Aniline can cause oxidative and nitrosative stress [3,4] and, ultimately, cancer in the spleens of rats. Aniline has also been found to be mutagenic, carcinogenic, and/or cytotoxic [2,5,6,7]. The toxicity and carcinogenicity of aniline in humans and animals have been well documented [8,9,10,11,12,13]. However, the molecular mechanism involved in aniline-induced liver toxicity and carcinogenesis remains unclear.

Mitochondria can convert 1%–2% of reduced oxygen into superoxide and they are the richest source of reactive oxygen species (ROS) in cells [14]. ROS produced by cells are involved in the regulation of different cellular functions, including intracellular signaling, transcription activation, proliferation, and apoptosis. In apoptosis, inhibition of the mitochondrial electron transport chain, resulting in the subsequent release of ROS, is an early event. ROS may be involved not only as specific messengers in the signal-transduction pathway but also as inducers of DNA damage in some cases of apoptosis [15]. 

The results of aniline exposure leading to toxicity of primary cultured hepatocytes and DNA damage have been observed in our [16] and other [17,18] previous studies. It has been clearly established that oxidative stress plays a role in the splenic toxicity of aniline, and aniline-induced oxidative stress is associated with increased oxidative DNA damage [19,20]. Cells respond to DNA damage by activating DNA-damage-inducible genes whose products contribute to cell cycle arrest and apoptosis [21]. Generation of ROS is one of the important biomarkers of cell proliferation and apoptosis [22]. However, it is unclear whether increasing ROS is a prerequisite for apoptosis and genotoxicity in hepatocytes. Therefore, the focus of the present study was to investigate the possible involvement of oxidative stress in the effects of aniline exposure in primary cultured hepatocytes.

## 2. Materials and Methods

### 2.1. Reagents

Aniline was from Demei Bio (Harbin, China). Neonatal calf serum and Dulbecco’s modified Eagle’s medium (DMEM) were from Imperial Laboratories (Dallas, TX, USA). Annexin V-FITC Apoptosis Detection Kit was from BD Biosciences (San Jose, CA, USA). The fluorescent probe 29, 79-dichlorodihydrofluorescein diacetate (DCFH-DA) was from Molecular Probes (Eugene, OR, USA). Other reagents were from Sigma (St. Louis, MO, USA).

### 2.2. Cell Culture

#### 2.2.1. Isolation of Rat Hepatocytes

Hepatocytes were isolated as described previously [23]. Male Sprague-Dawley rats (200–250 g) from College of Animal Science and Veterinary Medicine, Heilongjiang, China, were anesthetized with ether. The livers were perfused through the inferior vena cava with 200 mL solution A (0.14 M NaCl, 6.0 mM KCl, 1.5 mg/mL glucose, 0.5 pg/mL insulin, and 10 mM HEPES, pH 7.4) at 37 °C with a flow rate of 12 mL/min. This was followed by a 10–15 min perfusion with solution A containing 5.7 mM CaCl_2_ and 0.05% (*w*/*v*) collagenase (Type CLS, Cooper Biomedical, Palo Alto, CA, USA). The liver was removed, minced with scissors in solution A, and filtered through two layers of gauze. The cells were washed twice and then subjected to an iso-density Percoll centrifugation step to remove the damaged and dead hepatocytes. Viability of cells was >90% and there were 90 to 160 × 10^6^ cells/liver. All procedures were approved by the Ethics Committee for the Use of Experimental Animals in Harbin Medical University (No. HMUPHIRB2016003).

#### 2.2.2. Culture Conditions and Procedures

Hepatocytes (10^6^/mL) were plated in plastic culture dishes (Sangon Biotech Co. Ltd., Shanghai, China) with 3 mL (60 mm dishes) medium, which was a 1:1 mixture of DMEM (4.5 mg/mL glucose) and Ham’s F-12 with hypoxanthine (Hazelton Research Products, Lenexa, KS, USA). This medium was supplemented with gentamicin sulfate (25 μg/mL), amphotericin B (0.7 pg/mL), insulin (5 μg/mL from bovine pancreas; Sigma), and dexamethasone (10 μM) throughout the culture period. 

During the first 24 h, the medium contained 10% Nu-Serum (Collaborative Research, Bedford, MA, USA) and, thereafter, 5% Nu-Serum. The culture medium was replaced every 24 h, and the cultures were maintained at 37 °C in a humidified atmosphere containing 5% CO_2_ and 95% air. 

### 2.3. Measurement of Cell Viability by MTT Assay

The 3-(4,5-Dimethylthiazol-2-yl)-2,5-diphenyltetrazolium bromide (MTT) assay was used for the detection of cell viability [24,25,26]. Cells (10^5^) were incubated for 24 h at 37 °C in 0.5 mL medium. The cells were washed three times with medium and further incubated at 37 °C in 0.5 mL medium containing aniline (0, 1.25, 2.50, 5.0 and 10.0 μg/mL) for 20 h. Then, 20 μL phosphate-buffered saline containing MTT (Sigma) (5 mg/mL) was added, and the cells were further incubated for 4 h at 37 °C. After removal of the medium, 100 μL DMSO was added, and the absorbance at 550 nm was measured.

### 2.4. Detection of ROS

Cellular ROS levels were quantified as described previously [27]. Hepatocytes were seeded into six-well plates at a density of 5 × 10^5^/mL, cultured for 24 h, and exposed to aniline for 24 h in the presence or absence of *N*-acetyl-l-cysteine (NAC) (as mentioned above). The cells were incubated with DAFH-DA (final concentration, 10 M) for 15 min. Cells were washed with ice-cold phosphate-buffered saline (PBS), pH 7.4, collected, and kept on ice in the dark for immediate detection with a flow cytometer (FACS Calibur; BD Biosciences). H_2_O_2_ (20 μM) was used as the positive control.

### 2.5. Determination of Intracellular Total Glutathione (GSH)

The gutathione (GSH) assay kit (Jiancheng Bioengineering Co. Ltd., Nanjing, China) was used for determining GSH levels in the cell extracts, as described previously [28]. After treatment, hepatocytes were rinsed twice with PBS, and then collected in 500 μL PBS. One hundred microliters of cell suspension was treated with 5 μL 10% 5-sulfosalicylic acid and homogenized at 4 °C, followed by centrifugation at 20,000× *g* for 10 min. The supernatant fractions were analyzed with a glutathione (GSH) assay kit.

### 2.6. Detection of Superoxide Dismutase (SOD)

The method was based on the competition between superoxide dismutase (SOD) and tetrazolium blue for the superoxide radicals formed from the xanthine oxidase system [29,30]. After exposure to aniline for 24 h in the presence or absence of NAC, SOD activity was measured with a microplate spectrophotometer (Multiscan, Thermo Fisher Scientific, Waltham, MA, USA) at 550 nm.

### 2.7. Measurement of Catalase (CAT) Activity

CAT activity was measured with Aebi’s method [31]. Cells (10^5^) were exposed to aniline for 24 h in the presence or absence of NAC. Supernatant (0.1 mL) was added to a quartz cuvette containing 2.95 mL H_2_O_2_ solution (19.0 mmol/L) prepared in 0.05 M potassium phosphate buffer (pH 7.0). The change in absorbance was detected at 240 nm by using a Multiscan spectrophotometer (Thermo Fisher Scientific).

### 2.8. Membrane Lipid Peroxidation Assay

Malondialdehyde (MDA) represents the end product of lipid peroxidation [32]. The concentration of MDA can be measured by reacting with thiobarbituric acid (TBA) to form a stable chromophoric production. The MDA levels in the culture medium were measured by using an MDA assay kit (Jiancheng Bioengineering), as described previously [33]. The TBA method was used to analyze MDA by monitoring MDA-reactive products spectrophotometrically. The absorption was measured using a Multiscan spectrophotometer (Thermo Fisher Scientific) at 532 nm.

### 2.9. Detection of Mitochondrial Membrane Potential

Cells (10^5^) were exposed to aniline for 24 h in the presence or absence of NAC. The cytofluorometric analysis of mitochondrial membrane potential was evaluated with 5,5′,6,6′-tetrachloro, 1,1′,3,3′-tetraethylbenzimidazolylocarbocyanine iodide (JC-1; Invitrogen, Carlsbad, CA, USA), as described previously [34]. JC-1 accumulated in the mitochondrial matrix and showed green fluorescence with an emission wavelength of 525 nm (FL_1_), whereas, in the presence of high membrane potential, JC-1 showed red fluorescence with an emission length of 590 nm (FL_2_). Consequently, changes in mitochondrial membrane potential were determined using the FL_2_/FL_1_ ratio. 

### 2.10. DNA Damage Determination by Comet Assay

A comet assay was conducted to detect cellular DNA damage (single-strand breaks), as described previously [35]. Cells (10^5^) were exposed to aniline for 24 h in the presence or absence of NAC. H_2_O_2_ (20 μM; Demei Bio) was used as a positive control. Slides were viewed at 200× magnification using fluorescent microscopy. The excitation filter was 549 nm and the barrier filter was 590 nm. Comets were quantitatively analyzed using Comet Assay Software Project casp-1.2.2 (University of Wroclaw, Wroclaw, Poland). One hundred randomly selected cells from two microscope slides were analyzed and each treatment was carried out six times.

### 2.11. Assessment of Apoptosis

Apoptotic cell death was examined as described previously [36]. The apoptotic cell fraction in aniline-treated hepatocytes was assessed by flow cytometry. Cells (10^5^) were exposed to aniline for 24 h. In the 10.0 μg/mL + 20 mM NAC group, the hepatocytes were treated with NAC for 2 h before treatment with aniline. Quantification of apoptotic hepatocytes was performed with an Annexin V-FITC Apoptosis Detection Kit (BD Biosciences). At least 10,000 cells from each sample were examined using a FACS Calibur Analyzer (BD Biosciences). Experiments were repeated twice.

### 2.12. Statistical Analysis

Results were expressed as means and SDs. Statistical analysis was performed with one-way analysis of variance followed by Dunnett’s test. Differences were considered statistically significant at *p* < 0.05.

## 3. Results and Discussion

### 3.1. Aniline-Induced Generation of ROS and Oxidative Changes

**Figure 1:**
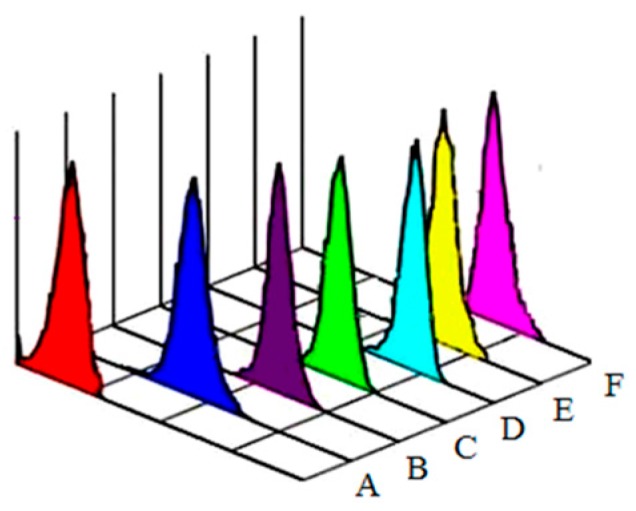
ROS levels of hepatocytes exposed to aniline. ROS levels gradually increased with concentration of aniline, in a dose-response relationship (A–D). A. 0 μg/mL; B. 2.50 μg/mL; C. 5.0 μg/mL; D. 10.0 μg/mL; E. 10.0 μg/mL + 20 mmol/L NAC; F. Positive control (H_2_O_2_).

Aniline is metabolized mainly in the liver [1]. The liver toxicity of aniline has been reported in epidemiological studies [37,38,39]. Aniline can lead to the formation of ROS, which induce oxidative stress [40,41]. Earlier studies have shown that aniline exposure is associated with oxidative stress in the spleen, as evident from the increases in lipid peroxidation and protein oxidation in that organ [19,42,43]. Excess generation of ROS results in oxidative stress which mediates apoptosis [44]. However, the mechanism of the induction of ROS by aniline remains unclear. We first investigated whether aniline stimulated ROS generation in hepatocytes. To accomplish this, we used DCFH-DA, the ROS-detecting fluorescent dye, to measure ROS production. The hepatocytes were exposed to 0, 2.50, 5.0 and 10.0 μg/mL aniline for 24 h, and ROS levels are shown in Figure 1A–D. ROS levels increased significantly with the concentration of aniline.

**Figure 2:**
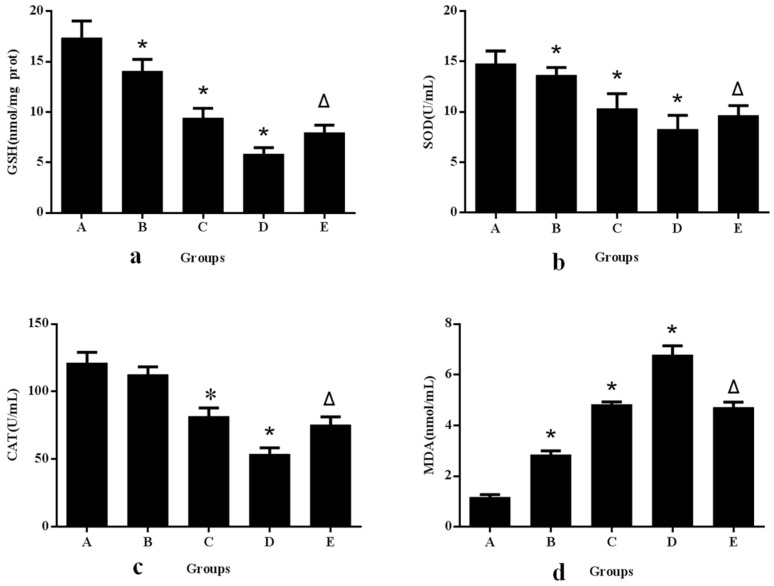
Changes in GSH, SOD, CAT and MDA levels after exposure of hepatocytes to aniline for 24 h. A. 0 μg/mL; B. 2.50 μg/mL; C. 5.0 μg/mL; D. 10.0 μg/mL; E. 10.0 μg/mL + 20 mmol/L NAC. Each data set mean value is a composite of three independent experiments with SD shown. * compared with control, *p* < 0.05; Δ compared with 10.0 μg/mL group, *p* < 0.05. (**a**) GSH; (**b**) SOD; (**c**) CAT; (**d**) MDA.

Furthermore, we evaluated the oxidative stress indexes including GSH, CAT, SOD and MDA in response to aniline exposure. The cellular antioxidant defense system relies on the endogenous antioxidants, such as GSH, SOD, and CAT. However, if ROS is generated at an inappropriate time or in excessive amounts, or if antioxidant defenses are overwhelmed, negative consequences of oxidative stress may occur. The levels of GSH, CAT, and SOD in the aniline groups were significantly decreased and the production of MDA was increased compared to those in the control group (Figure 2a–d), which were indicators of lipid peroxidation. We confirmed that aniline increased the formation of ROS and induced oxidative stress and apoptosis. A previous study has shown that the increase in cytosolic and mitochondrial SOD could be a defensive adaptation to handle oxidative stress, as reported for aniline toxicity in rabbit lung [45]. Khan et al. have evaluated markers of oxidative stress such as lipid peroxidation, GSH, and NO. The aniline hydrochloride (AH)-induced group showed a significant increase in lipid peroxidation and NO (which forms a part of the reactive nitrogen species), whereas there was a significant decrease in the GSH level in the spleen and liver [46]. Our results are also in agreement with a previous study that indicated that oxidative stress contributes to the mechanism of cell death with aniline and its compounds. Splenotoxicity of aniline is associated with iron overload and ROS, which can cause oxidative damage to DNA, proteins and lipids (oxidative stress) [20]. 

### 3.2. Aniline Causes Loss of Mitochondrial Permeability

**Figure 3:**
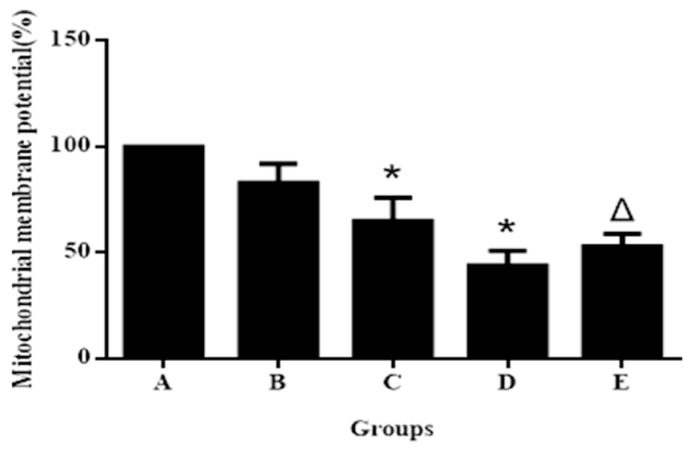
Mitochondrial membrane potential of hepatocytes exposed to aniline for 24 h. Data represented are mean ± SD of three identical experiments made in triplicate. A. 0 μg/mL; B. 2.50 μg/mL; C. 5.0 μg/mL; D.10.0 μg/mL; E. 10.0 μg/mL + 20 mmol/L NAC. * Compared with control, *p* < 0.05; Δ compared with 10.0 μg/mL group, *p* < 0.05.

Generation of intracellular ROS may be related to mitochondrial dysfunction and induction of apoptosis in various cell types. We found that the mitochondrial membrane potential in hepatocytes treated with 2.50, 5.0 and 10.0 μg/mL aniline for 24 h decreased significantly in a concentration-dependent manner (Figure 3). This indicated that aniline could also cause the loss of mitochondrial permeability.

### 3.3. Aniline-Induced DNA Injury in Hepatocytes

Recent reports have emphasized the role of nuclear DNA damage in apoptosis induced by ROS [47]. We used the comet assay to examine DNA damage in response to aniline to explore the mechanism of apoptotic cell death induced by aniline. It is well known that various toxic agents can induce DNA damage. DNA fragmentation is a biochemical hallmark of apoptosis. Induction of apoptosis has been recognized as a possible outcome of DNA damage for >35 years [48]. The comet assay is increasingly accepted by regulatory agencies for use in evaluating the genotoxic potential of chemicals. DNA damage in hepatocytes exposed to aniline was estimated as arbitrary units by the comet assay. The arbitrary unit was selected for evaluation because it was considered a sensitive measure of DNA damage, based on the length of migration and the amount of DNA in the tail [49]. 

**Figure 4:**
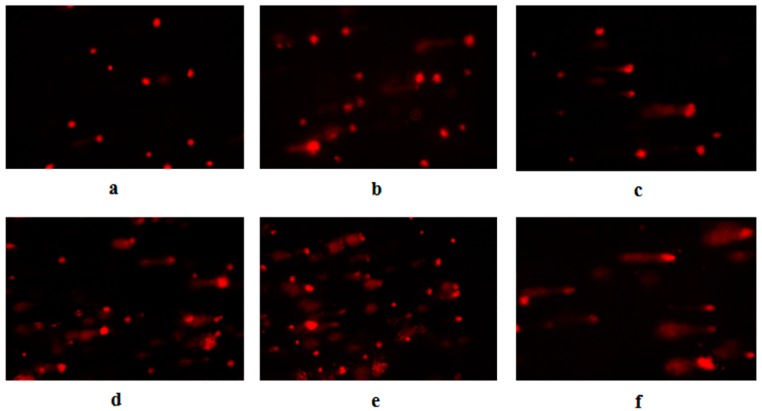
The degree of DNA damage of hepatocytes exposed to aniline for 24 h. (**a**) 0 μg/mL; (**b**) 2.50 μg/mL; (**c**) 5.0 μg/mL; (**d**) 10.0 μg/mL; (**e**) 10.0 μg/mL + 20 mmol/L NAC; (**f**) positive control (H_2_O_2_). (Original magnification 100×).

Aniline can induce two well-documented cellular changes, oxidative stress and DNA damage, which contribute to carcinogenesis and toxicity [28,50]. Our results demonstrated that aniline significantly induced DNA damage as evident by the olive tail moment (Figure 4a–d and Table 1). Under the fluorescence microscope, hepatocytes in the negative control group were round with no comet formation. Comet tails were observed in hepatocytes treated by aniline. The tail moment and DNA content of the tail increased with the concentration of aniline. Compared with those in the negative control group, the tail moment and DNA content of the tail were significantly higher (*p* < 0.05), which indicated that aniline could induce DNA damage. 

### 3.4. ROS Generation and DNA Injury Play a Role in Analine-Induced Apoptosis in Hepatocytes

**Figure 5:**
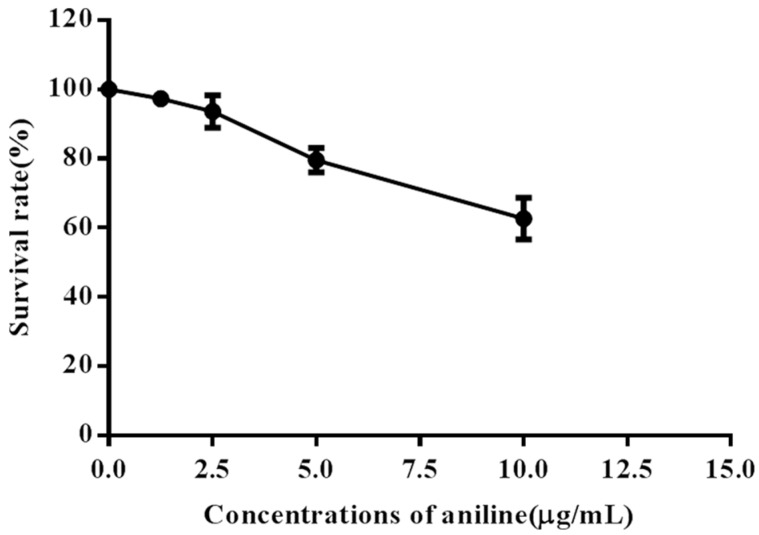
Viability of hepatocytes exposed to aniline. Hepatocytes were cultured in the absence or presence of 1.25, 2.5, 5.0 or 10.0 μg/mL aniline for 24 h. Cell viability was determined based on the MTT assay. Data represented are mean ± SD of three identical experiments made in triplicate.

We evaluated the effects of aniline on cell viability using the MTT assay. Aniline decreased cell viability in a concentration-dependent manner (Figure 5). The MTT assay showed that 5.0–10.0 μg/mL aniline suppressed hepatocyte growth, with a 20%–37% reduction as compared to the negative control group. 

**Figure 6:**
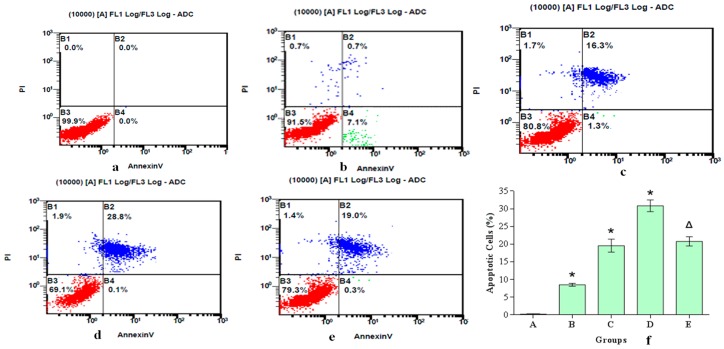
Apoptosis of hepatocytes exposed to 0, 2.50, 5.0 or 10.0 μg/mL aniline for 24 h. Cells were treated with aniline, then stained with fluorescein isothiocyanate-conjugated Annexin V and propidium iodide using flow cytometry. (**a**) 0 μg/mL; (**b**) 2.50 μg/mL; (**c**) 5.0 μg/mL; (**d**) 10.0 μg/mL; (**e**) 10.0 μg/mL + 20 mmol/L NAC; (**f**) Apoptotic cells (%). Data represented are mean ± SD of three identical experiments made in triplicate. * Compared with control, *p* < 0.05; Δ compared with 10.0 μg/mL group, *p* < 0.05.

To determine whether the decrease in hepatocyte viability in response to aniline treatment was due to the induction of apoptosis, flow cytometry was used to detect apoptotic cell death. Aniline induced apoptotic cell death in a concentration-dependent manner (Figure 6a–d). These findings suggest that hepatocytes undergo apoptosis and growth inhibition after exposure to aniline. 

To establish whether the generation of ROS resulted in oxidative stress involved in aniline-induced cellular injury or apoptosis, we used the antioxidant NAC to decrease the superoxide levels induced by aniline. The addition of NAC, an ROS inhibitor, before aniline treatment prevented ROS generation (Figure 1E), oxidative changes (Figure 2E), loss of mitochondrial membrane potential (Figure 3E), and apoptosis (Figure 6e). Compared with the 10.0 μg/mL aniline group, the ROS level in the aniline + NAC group was decreased by 29.7%, and the levels of GSH, SOD and CAT in the aniline + NAC group were increased by 38.6%, 19.8% and 42.1%, respectively. The level of MDA in the aniline + NAC group was decreased by 38.3%. The results indicated that the oxidative changes induced by aniline were significantly blocked by pretreatment with NAC. The addition of NAC prior to aniline treatment increased the mitochondrial membrane potential and blocked apoptosis of hepatocytes, compared with the 10.0 μg/mL aniline group. Hepatocyte apoptosis decreased by 29.8% and the mitochondrial membrane potential increased by 34.8%. The results showed that aniline-induced loss of mitochondrial membrane potential via oxidative stress was significantly blocked by NAC pretreatment.

In aerobic cells, ROS are generated as a by-product of normal mitochondrial activity. If not properly controlled, ROS can cause severe damage to cellular macromolecules, especially DNA. Intracellular generation of ROS is a crucial factor in the apoptotic pathway, as well as in DNA damage, including oxidized base lesions, single- and double-strand breaks, and many other cellular processes that could be cytotoxic, genotoxic or mutagenic [20]. Here, we provided evidence that aniline might lead to DNA damage in hepatocytes. The addition of NAC decreased ROS generation, and the number and tail length of comet cells were significantly lower than in the 10.0 μg/mL aniline group. These data suggest that ROS generation is involved in the loss of mitochondrial membrane potential and DNA injury, which may play a role in aniline-induced apoptosis of hepatocytes. 

## 4. Conclusions

The present study demonstrates that aniline exposure induces ROS which increase lipid hydroperoxide levels, loss of mitochondrial membrane potential, and DNA damage, and which can lead to changes in hepatocyte viability and apoptosis. In summary, these findings suggest that oxidative stress plays a role in aniline-induced cyto/genotoxicity and apoptosis of hepatocytes. Time course data are needed to fully elucidate the mechanism of aniline toxicity in hepatocytes.

## Figures and Tables

**Table 1:** Degree of DNA damage in hepatocytes exposed to aniline for 24 h.

Number	Group	Amount of Cells	Amount of Comet Cells	Percent of Comet Cells (%)	Length of Comet Tail (μm)
1	0 μg/mL	600	23	3.83 ± 1.17	12.02 ± 1.79
2	Positive control	600	583	97.17 ± 1.83	43.58 ± 6.77
3	2.50 μg/mL	600	64	10.67 ± 4.59 *	16.54 ± 3.27 *
4	5.00 μg/mL	600	127	21.17 ± 2.64 *	28.67 ± 5.01 *
5	10.00 μg/mL	600	214	35.67 ± 4.89 *	41.24 ± 6.12 *
6	10.00 μg/mL+	600	142	23.67 ± 3.67 *^,Δ^	30.46 ± 5.15 *^,Δ^
	20 mM NAC				

Results shown as mean ± SD. Aniline-induced DNA damage. Comets were quantitatively analyzed using Comet Assay Software. One hundred randomly selected cells from two microscope slides were analyzed and each treatment was carried out six times. * Compared with control, *p* < 0.05; ^Δ^ compared with 10.0 μg/mL group, *p* < 0.05.
